# Reply to Castro et al.: Do connectomes possess markers of activity-dependent synaptic plasticity?

**DOI:** 10.1073/pnas.2317056120

**Published:** 2023-12-04

**Authors:** Nikolai M. Chapochnikov, Cengiz Pehlevan, Dmitri B. Chklovskii

**Affiliations:** ^a^Department of Neurology, New York University School of Medicine, New York, NY 10016; ^b^John A. Paulson School of Engineering and Applied Sciences, Harvard University, Cambridge, MA 02138; ^c^Center for Brain Science, Harvard University, Cambridge, MA 02138; ^d^Kempner Institute for the Study of Natural and Artificial Intelligence, Harvard University, Cambridge, MA 02138; ^e^Center for Computational Neuroscience, Flatiron Institute, New York, NY 10010; ^f^Neuroscience Institute, New York University School of Medicine, New York, NY 10016

We introduced a model of an olfactory microcircuit of the Drosophila larva (1) and proposed that synaptic weights could arise via genetic encoding or activity-dependent (e.g., Hebbian) plasticity. Inspired by Motta et al. (2), Castro et al. (3) tested for activity-dependent synaptic plasticity in the circuit by analyzing a “point-in-time” snapshot: the connectome reconstruction (4).

We refute the hypotheses and analysis of Castro et al. (3). Foremost, Motta et al. only proposed a tentative explanation for their empirical observations rather than a test for activity-dependent plasticity (2). The Letter hypothesizes that after LTP (LTD) in a synaptic connection, the median synaptic size increases (decreases) and the median absolute deviation (MAD) of synaptic sizes decreases (increases). Thus, the correlation coefficient between the median and the MAD would be negative in a set of selected synaptic connections that underwent plasticity, which they did not observe. They seem to assume that the correlation coefficient starts at approximately 0 and becomes negative after plasticity, thus making the coefficient informative about recent synaptic plasticity. Our analysis and numerical simulation refute these assumptions (5). We show that the correlation coefficient for a set of synapses can initially be positive and does not necessarily decrease or turn negative after synaptic plasticity, rendering this measure uninformative per se.

**Fig. 1.**
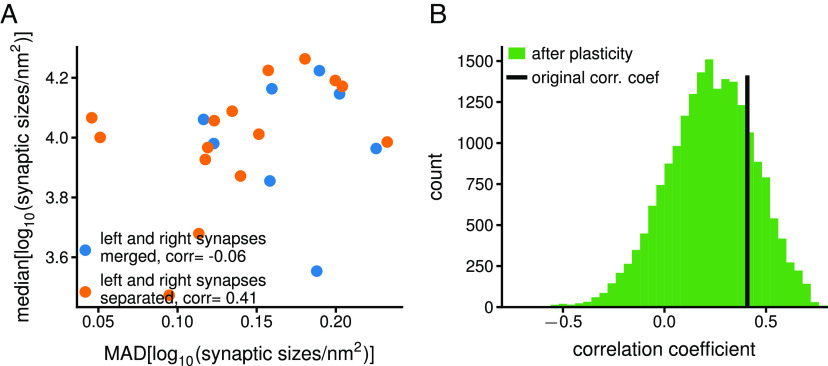
Correlation coefficient between the median and the MAD of synaptic sizes and simulation of plasticity. (*A*) *Blue*: same analysis as in figure 1B of ref. 3, when merging the synaptic connections on the left and right side of the larva (resulting in 8 “synaptic connections”). *Orange*: analyzing the left and right synaptic connections separately (16 synaptic connections in total, only 15 shown because 1 synapse with a single synaptic contact was excluded). The correlation coefficient is between the median of the log10 of the synaptic sizes and the MAD of the log10 of the synaptic sizes per synaptic connection. (*B*) Histogram of correlation coefficients resulting from numerical simulations. Starting with the 15 synaptic connections from (*A*), in each simulation run, each connection undergoes either LTP (probability = 0.5) or LTD (probability = 0.5). When a synaptic connection undergoes LTP (LTD) the size of all the synaptic contacts is doubled (halved). 20,000 simulation runs, bin size: 0.04.

We replicate figure 1B of ref. 3, but without merging the synaptic connection of the left and right side of the larva, which seems unsuitable, since it combines synaptic sizes from different connections. Following (3), we compute the median and the MAD of the logarithm of synaptic sizes for each synaptic connection, and obtain a correlation coefficient of 0.41 for the 15 synaptic connections (excluding one synapse with a single synaptic contact), different from the −0.06 obtained for 8 merged connections (Fig. 1*A*). Notably, when selecting only the 7 connections with LNs, it becomes 0.06, highlighting this measure’s sensitivity to connection selection.

We then simulate strong LTP and LTD on the set of 15 synaptic connections by randomly doubling or halving the synaptic sizes in each synaptic connection. We bound the synaptic sizes between the minimum and the maximum sizes found in all these synapses (924 nm^2^ and 61,400 nm^2^). We compute the resulting correlation coefficients between the median and the MAD (Fig. 1*B*). In 21% of cases, the coefficient increased, contradicting the assumed decrease with synaptic plasticity. In 85% of cases, it remained positive, demonstrating that a negative coefficient is not necessary for recent plasticity.

Furthermore, this approach overlooks potential changes in synaptic contact number during synaptic plasticity, which would further complicate the interpretation of the correlation coefficient and of synaptic size over-similarity (3). Given the wide variation of synaptic contact number (from 1 to 80 synaptic contacts per connection in ref. 4), changes in synaptic number undoubtedly play a role in plasticity.

In conclusion, we argue this method is not viable for detecting activity-dependent plasticity. The question of activity-dependent plasticity in this circuit remains open, and we hope that it will be answered by developing new markers in electron microscopy.
